# Influence of adjuvant antibiotics on fistula formation following incision and drainage of anorectal abscesses: a systematic review protocol

**DOI:** 10.1186/s13643-019-1002-z

**Published:** 2019-04-15

**Authors:** Laura Baker, Lara Williams, Remington Winter, Caitlin Cahill, Alexandra Davis, Dean Fergusson

**Affiliations:** 1 Department of Colorectal Surgery, Division of Surgery, The Ottawa Hospital, Ottawa, Canada; 2 Clinical Epidemiology Program, The University of Ottawa, Ottawa, Canada; 3 Department of Surgery, University of Ottawa, Ottawa, Canada; 4 Faculty of Medicine, The University of Ottawa, Ottawa, Canada; 5 Library Services, The Ottawa Hospital, Ottawa, Canada; 6 Ottawa Hospital Research Institute, Ottawa, Canada

**Keywords:** Fistula, Abscess, Anti-bacterial agents, Anal Canal, Rectum

## Abstract

**Background:**

Development of fistula-in-ano following incision and drainage (I&D) of anorectal abscesses occurs in over 30% of patients. It is associated with significant patient morbidity and societal cost. The use of antibiotics following drainage is controversial, with randomized controlled trials reporting opposing conclusions regarding their influence on the rate of fistula formation. Given the significant burden associated with their development, it is imperative to determine strategies to minimize their occurrence. The objective of this review is to summarize the available evidence on the role of antibiotics following I&D of anorectal abscesses on fistula formation. Secondary objectives include determining if antibiotics are associated with morbidity, repeat presentation to the emergency department, and requirement for reoperation.

**Methods/design:**

MEDLINE, EMBASE, CINAHL, Cochrane Central Registry of Controlled Trials, http://apps.who.int/trialsearch, and clinicaltrials.gov will be searched to identify published and ongoing unpublished interventional and observational studies evaluating the role of antibiotics post I&D on the incidence of fistula formation. There will be no restriction on language, date, or journal. Title and abstracts as well as full texts will be screened in duplicate based on inclusion and exclusion criteria. The Cochrane Risk of Bias tool and ROBINS-I will be used to assess risk of bias in randomized and non-randomized studies, respectively. Our primary outcome is the incidence of fistula formation; secondary outcomes include morbidity, representation to ED, and reoperation. Study heterogeneity will be calculated with Cochran’s *Q* test, *P* value, and *I*^*2*^ index. SASS (version 9.4) will be used for meta-analysis.

**Discussion:**

This is the first study to review the available evidence on adjuvant antibiotics and incidence of fistula formation following I&D of anorectal abscesses.

**Systematic review registration:**

PROSPERO CRD42018092044

**Electronic supplementary material:**

The online version of this article (10.1186/s13643-019-1002-z) contains supplementary material, which is available to authorized users.

## Background

Perianal fistula formation following drainage of anorectal abscesses is common and associated with significant morbidity. They result from the formation of an epithelized tract between the anal canal and perianal skin, most commonly following infection originating from an anal gland [1]. Perianal fistulas reportedly develop in approximately 37% of patients who undergo incision and drainage (I&D) of anorectal abscesses [2, 3]. They are associated with pain, malodorous perianal drainage, increased health care resource utilization (emergency department visits, hospital admissions), and decreased quality of life [4, 5]. Medical and surgical therapies are often indicated in the management of perianal fistulas [6]. Given their associated morbidity, mechanisms to decrease the development of perianal fistulas are essential.

Antibiotic administration following I&D of perianal abscesses has been proposed; however, the literature on the subject is conflicting and their role is not well defined. Some advocate for prophylactic postoperative antibiotics following I&D [7, 8]. One hypothesis is that antibiotics aid in the eradication of residual infection, which is what is thought to be responsible for the formation of a fistula tract [9]. Others suggest limiting antibiotic administration to patients who are immunocompromised, are diabetic, have artificial cardiac valves, or are found to have extensive cellulitis on physical exam at the time of presentation [7]. Randomized trials investigating the role of adjuvant antibiotics on fistula formation following I&D of perianal abscesses report conflicting results. The study by Ghahramani et al. favored a 7-day course of adjuvant antibiotics (*n* = 307), whereas Sozener et al. favored placebo to a 10-day course of antibiotic (*n* = 183) [10, 11]. In summary, there is a lack of consensus in the literature regarding the role of adjuvant antibiotics following I&D of perianal abscesses.

A systematic review of the literature evaluating the impact of adjuvant antibiotic therapy on perianal fistula development does not currently exist. Given the controversy on the subject, we propose a systematic review of the literature evaluating the influence of antibiotic administration following I&D of perianal abscesses on the incidence of fistula formation. Recommendations generated from this review will help inform future practice or need for further investigation.

### Objective

The primary objective of this review is to determine if antibiotic administration following I&D of anorectal abscesses influences the incidence of fistula formation based on the results of interventional and observational studies. Secondary objectives will be to (1) determine if adjuvant antibiotics influence the incidence of morbidity, presentation to the emergency department, and reoperation and (2) evaluate the quality of evidence of included articles.

## Methods

The Preferred Reporting Items for Systematic Review and Meta-analysis Protocols (PRISMA-P) checklist guidelines were referenced to achieve the highest standard in reporting items for a systematic review and meta-analysis [12, 13]. The PRISMA-P checklist is included in Additional file 1. The PRISMA flow diagram will be utilized to display the screening strategy. The protocol was registered with the PROSPERO International Prospective Register of Systematic Reviews on March 5, 2018 (CRD42018092044).

Any amendments made to the current protocol will be published using a protocol addendum, accompanied by the date of and rationale for the reported amendment, with the final manuscript.

### Eligibility criteria

#### Study designs

Given the assumed paucity of published studies evaluating the effectiveness of antibiotic therapy for the prevention of perianal fistula following I&D, we will purposely leave our study design criteria broad to get a comprehensive picture of the evidence to date. We will include all published interventional and observational studies that report an association between perianal fistula and antibiotics following I&D as a primary objective of the study. Case reports reporting on one individual will be excluded from the review.

#### Population

Patients of all ages presenting with anorectal abscesses (inclusive of perianal, ischiorectal, intersphincteric, supralevator, and horseshoe abscesses), of any etiology with the exception of Crohn’s disease, undergoing I&D will be included. Patients in whom a fistula is identified at the time of initial I&D will be excluded. Patients with Crohn’s disease will be excluded as they are routinely treated with antibiotics and are at increased risk of developing a perianal fistula in comparison to those without [14]. Patients presenting with pilonidal and scrotal abscesses or infected Bartholin cysts will be excluded.

#### Intervention

The intervention under investigation is antibiotic administration following I&D of anorectal abscesses. There is no minimal duration of adjuvant antibiotic therapy. Both oral and intravenous administration of antibiotics will be included.

#### Comparators

The comparator group will be individuals that did not receive antibiotics following surgical drainage of perirectal abscesses.

### Outcomes

#### Primary outcome

The primary outcome is the incidence of fistula formation following I&D of anorectal abscess. The diagnosis of fistula-in-ano can be made based on clinical assessment (inclusive of physical exam) or radiographically by cross-sectional imaging or ultrasound. If the primary outcome is assessed at multiple time points, we will collect all documented outcomes.

#### Secondary outcomes

Secondary outcomes include post procedural adverse events, presentation to the emergency department, incidence of reoperation, and subsequent procedural management in the event a fistula develops as well as patient-reported quality of life. Adverse events will be evaluated as per the Clavien Dindo Classification System [15]. Morbidity suspected to have resulted from antibiotic administration (for example, adverse reaction or *Clostridium difficile* infection) will also be recorded. Emergency room visits following initial I&D procedure will encompass all subsequent visits with the presenting or chief complaint being related to their anorectal abscess. Reoperation is defined by repeat I&D at the site of original drainage. It includes procedures performed both at the bedside and in the operating room. Procedural management of a fistula includes, but is not limited to, seton placement, fistulotomy, advancement flaps, use of fibrin sealant, or fistula plug or ligation of the intersphincteric fistula tract. Reporting of quality of life with a validated questionnaire will be included as a secondary outcome. Similar to the primary outcome, we will extract all outcomes of interest and their reported timing from incision and drainage.

The quality of evidence will also be evaluated through assessment of risk of bias.

#### Study type

All studies meeting inclusion criteria will be considered, regardless of study duration, language of publication, sample size, or geographic location. Both published and unpublished data will be included.

### Search strategy

A systematic search of electronic databases will be performed to identify all relevant studies investigating the association between adjuvant antibiotic therapy and incidence of fistula-in-ano. A reference librarian was consulted to assist with the development of database-specific search strategies. We used exploded Medical Subject Headings (MeSH) and keywords to search the following themes: abscess, anal canal, rectum, drainage, and anti-bacterial agents (a draft of the MEDLINE search strategy is included in Additional file 2). The search strategy will be reviewed by a second medical librarian using the Peer Review of Electronic Search Strategies (PRESS) checklist.

We will apply the search strategy to the following databases: MEDLINE (PubMed, PubMed in Process, and Ovid), EMBASE, CINHAL, and Cochrane Central Registry of Controlled Trials. Additionally, http://apps.who.int/trialsearch and clinicaltrials.gov will be searched for unpublished in-progress studies. OpenGrey will also be queried for grey literature of relevance. References of included manuscripts will be reviewed to identify additional studies of relevance.

### Study selection

Articles identified through the search strategy will be imported into Covidence, an online citation manager [16]. Abstracts and titles, followed by full manuscripts, will be screened through the Covidence Platform. Team members involved in article screening will receive training on the program (LB, RW, and LW).

**Fig. 1:**
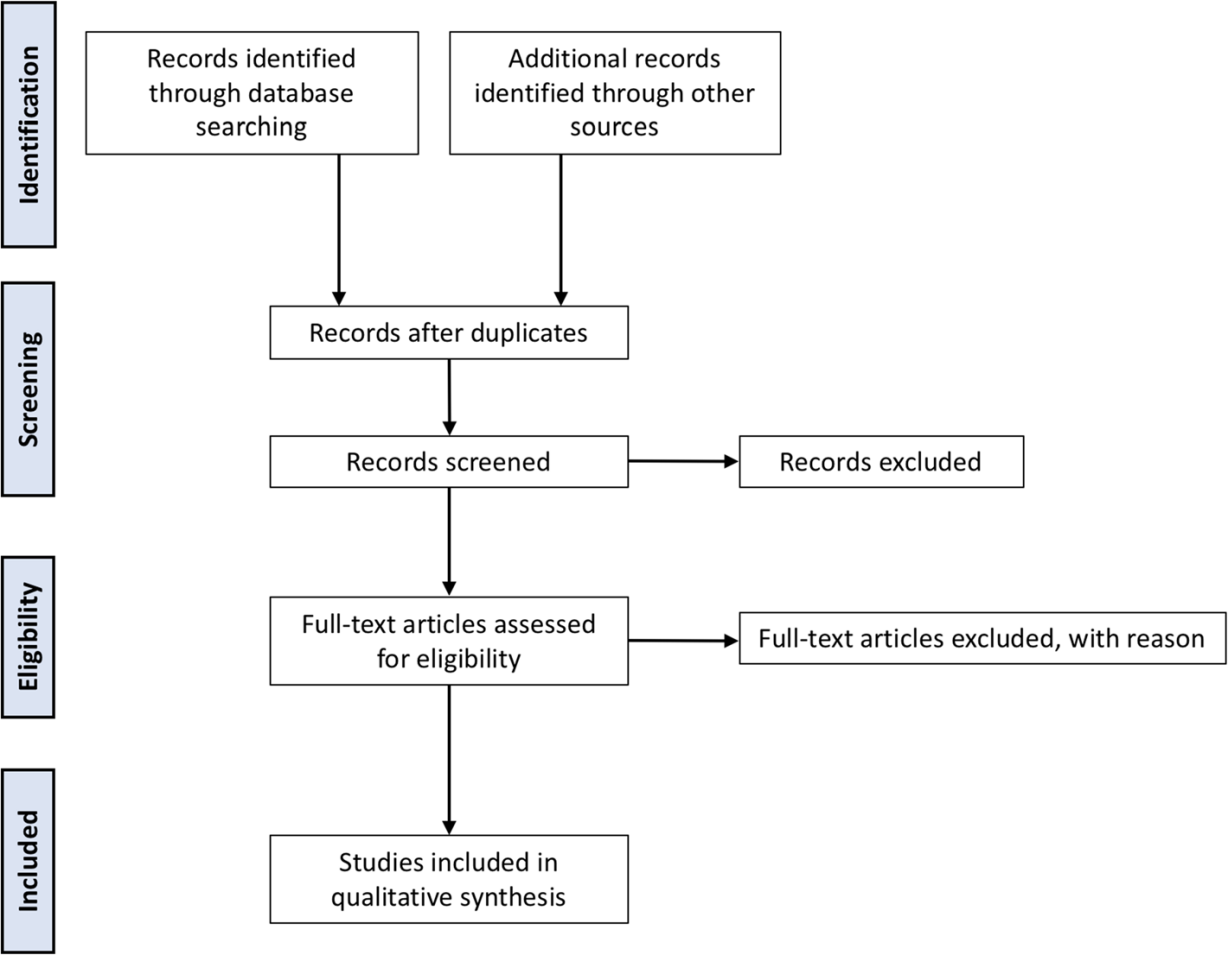
PRISMA flow diagram

All titles and abstracts identified will be independently screened by two reviewers (LB and RW) for relevance and categorized as relevant, possibly relevant, or irrelevant. Manuscripts of articles categorized as relevant or possibly relevant will be retrieved for further evaluation. Full texts will also be reviewed in duplicate for eligibility (LB and RW). Any disagreement regarding relevancy will be resolved by the senior author (LW). Reason for study exclusion will be documented and presented in the PRISMA flow diagram for study screening (Fig. 1).

### Data extraction

Data extraction will be conducted by two reviewers (LB and RW) using a standardized electronic data extraction form. The data extraction form will be piloted by both reviewers. Data extraction will be performed independently, in duplicate. The following information will be extracted from each article: study identifiers (title, authors, journal, publication date, study location(s), funding), aspects of study design (interventional vs observational, blinding, allocation concealment, duration, setting, number of centers), patient characteristics (inclusion/exclusion criteria, baseline demographics), intervention information (interventions comparing adjuvant antibiotics vs no adjuvant antibiotics, antibiotic class and dose prescribed, and route of administration), outcome (fistula formation, adverse event, representation to ED, reoperation for recurrent abscess, additional procedural management of fistula), and patient-reported quality of life.

In the event data pertaining to treatments or outcomes of interest are missing, authors will be contacted in an attempt to retrieve additional information. If the corresponding authors neglect to respond within a 1 week period, they will be contacted two additional times. If contact information is not available, authors will not be attempted to be contacted.

In the event studies referring to the same patient population are identified (duplicate, overlapping, or companion studies), only the most recent or comprehensive study will be included.

### Risk of bias/quality assessment

The risk of bias of observational and interventional studies will be evaluated by two independent assessors (LB and RW) using the Cochrane Collaboration’s tool for assessing risk of bias in randomized controlled trials (RCTs) and ROBINS-I tool for non-RCTs [17, 18].

### Data synthesis

A descriptive summary table of primary and secondary outcomes will be presented for all included studies. A direct meta-analysis will be performed for primary and secondary outcomes of randomized and quasi-randomized studies. The incidence of fistula formation, major postoperative morbidity (Clavien Dindo Classification 3 or greater), and requirement of reoperation or additional procedural management will be analyzed as dichotomous data, with risk ratios with 95% confidence intervals (CI). The number of emergency room visits, a continuous outcome, will be analyzed using weighted mean differences with 95% CI. The random effects model will be used to generate forest plots. Publication bias will be evaluated through generation of funnel plots of standard error against the log odds ratio. *P* < 0.05 will be considered statistically significant for all analyses.

Heterogeneity between studies will be tested with the chi-square test (significance level 0.1) and *I*^2^ statistic. *I*^2^ above 50% will be considered substantial heterogeneity.

Statistical analyses will be conducted, and figures will be generated by the software RevMan 5.3 [19].

Studies will be grouped into the following subgroups for further analysis: patient age (pediatric versus adult), study design, antibiotic used, and underlying pathology of anorectal abscess.

If significant heterogeneity is identified, sensitivity analysis will be performed to explore potential sources of heterogeneity. Sensitivity analysis excluding articles at high risk of bias, unpublished articles, and abstract publications (compared to full-text publications) will be conducted.

The Grading of Recommendations Assessment Development and Evaluation working group methodology (GRADE) will be applied to evaluate the confidence of the level of evidence [20].

## Discussion

Lack of agreement regarding the role of antibiotics following I&D of anorectal abscesses currently exists. Given the simplicity of the proposed intervention and the high degree of morbidity associated with the development of the outcome of interest, perianal fistula, establishing if there is a possible benefit is critical. The results of this review have the potential to influence the management of one of the most commonly encountered benign perianal pathologies.

Not only will the proposed review summarize the influence of adjuvant antibiotics on the development of perianal fistula, but also the analysis of secondary outcomes will contribute to our understanding of whether antibiotics decrease the economic burden and impairment in quality of life associated with anorectal abscesses.

The strengths of the proposed review are the broad search strategy as well as the planned subgroup analyses. In the event meta-analysis is possible, results of this study will allow generation of recommendations regarding management. This review is anticipated to be limited by a lack of RCTs and study heterogeneity.

Potential challenges include our ability to draw conclusions from pooled trials if significant heterogeneity exists between studies. In an attempt to mitigate the influence of heterogeneity, we plan on limiting meta-analysis to randomized and quasi-randomized studies. Additionally, if the findings permit, we plan on conducting further subgroup analysis, specifically type of perianal abscess, class of antibiotics, and duration of antibiotics. In the event subgroup analysis is not possible, we will be conservative with the reported conclusions, highlighting the limitations of the study.

In conclusion, a systematic review and meta-analysis of the literature pertaining to the role of antibiotics in the treatment of anorectal abscesses following I&D will allow us to generate recommendations on the management of these patients and, in the event data is lacking, will inform if there is a need for future investigation.

## Additional files

Additional file 1: PRISMA-P Checklist (See additional document). (DOCX 30 kb)

Additional file 2: Medline search strategy (DOCX 13 kb)

## Abbreviations

I&D: Incision and drainage
PRESS: Peer Review of Electronic Search Strategies
PRISMA-P: The Preferred Reporting Items for Systematic Review and Meta-analysis Protocols
RCT: Randomized controlled trial
ROBINS-I: Risk of bias in non-randomized studies of interventions
