# Supplementary material for: Influence of adjuvant antibiotics on fistula formation following incision and drainage of anorectal abscesses: a systematic review protocol
Source: Syst Rev. 2019 Apr 15;8:95. doi: 10.1186/s13643-019-1002-z (PMC6463650; doi:10.1186/s13643-019-1002-z)
Supplement: Supplementary file 2 — Medline search strategy (DOCX 13 kb) [file 13643_2019_1002_MOESM2_ESM.docx]

Medline search strategy

**Database: Ovid MEDLINE(R) Epub Ahead of Print, In-Process & Other Non-Indexed Citations, Ovid MEDLINE(R) Daily and Ovid MEDLINE(R) <1946 to 2018 March 12>
Search Strategy:**--------------------------------------------------------------------------------
1     ABSCESS/ or abscess*.tw,kw. (76139)
2     Anus Diseases/ (4067)
3     ANAL CANAL/ or Rectum/ (48791)
4     (perianal or peri-anal or anoperineal or ano-perineal or anorectal or perirectal or peri-rectal or anus or anal or supralevator or ischiorectal or intersphincter*).tw,kw. (50408)
5     2 or 3 or 4 (84931)
6     1 and 5 (2875)
7     ((rectal or rectum) adj2 abscess*).tw. (99)
8     Drainage/ or (drain* or incision* or postoperative* or post-operative*).tw,kw. (665163)
9     (6 or 7) and 8 (1151)
10     Abscess/su (4793)
11     10 and 5 (437)
12     9 or 11 (1351)
13     exp Anti-Bacterial Agents/ (645900)
14     (antibiotic* or anti biotic* or anti bacterial agent* or antibacterial agent* or antibacterial drug* or anti bacterial drug*).tw,kw. (306086)
15     Antibiotic Prophylaxis/ (12295)
16     13 or 14 or 15 (779480)
**17     12 and 16 (242)**
